# Seven draft genomes of *Kluyveromyces marxianus* strains from alcoholic fermentations in South Africa

**DOI:** 10.1128/mra.01042-25

**Published:** 2026-01-29

**Authors:** Jesús Martín Moreno-Hernández, Luis F. García-Ortega, Lucia Morales, Carolina Henritta Pohl, Alexander DeLuna, Eugenio Mancera

**Affiliations:** 1 Departamento de Ingeniería Genética, Unidad Irapuato, Cinvestav 98805 , Irapuato, Mexico; 2 Unidad de Genómica Avanzada, Cinvestav, Irapuato, Mexico; 3 Laboratorio Internacional de Investigación sobre el Genoma Humano (LIIGH), Universidad Nacional Autónoma de México 7180, Juriquilla, Mexico; 4 Department of Microbiology and Biochemistry, University of the Free State 37702, Bloemfontein, South Africa; 5 Centro de Investigación sobre el Envejecimiento, Cinvestav 505469 , Ciudad de México, Mexico; University of California Riverside, Riverside, California, USA

**Keywords:** *Kluyveromyces marxianus*, genome drafts, South Africa, beer, agave spirits

## Abstract

Despite its recognized potential for industrial biotechnology and lactose-based fermentations, the genomic diversity of the yeast *Kluyveromyces marxianus* remains underexplored, especially in non-dairy contexts. Here, we report the draft genomes of seven strains isolated from South Africa, expanding the known genetic diversity of this versatile species.

## ANNOUNCEMENT

*Kluyveromyces marxianus* is a yeast with broad biotechnological potential, including applications in fermented foods and chemical biomanufacturing. As an emerging model in synthetic biology, successful strain engineering depends on the availability of whole-genome sequences for many genetic backgrounds (1, 2). Therefore, exploring its genomic diversity is essential to fully uncover its applied potential. Only 35 *K*. *marxianus* genomes are currently available at NCBI; despite this limited sampling, the species exhibits high genetic diversity, nearly threefold that of *Saccharomyces cerevisiae*, for which thousands of genomes are available (3). Here, we report the draft genomes of seven strains from traditional beer fermentation, soil, and agave spirits production (four, one, and two isolates, respectively) in South Africa, expanding the resources for biotechnology research.

The seven strains were obtained from the Biodiversity Biobanks South Africa, Yeast Culture Collection at the University of the Free State, Bloemfontein, South Africa. Originally, they were isolated by diluting the substrate in PBS and plating onto yeast-malt extract agar containing tartaric acid (pH 4, 30°C). DNA was purified from overnight YPD cultures (30°C) using phenol–chloroform (4) and quantified with a Qubit fluorometer. Libraries were prepared with the MGIEasy FS DNA Library Prep kit and sequenced using the DNBSEQ platform by the Beijing Genomics Institute-BGI (Guangdong, China). Base calling was performed using Zebracall (BGI), which automatically converts raw signal data into FASTQ files (5, 6). Sequencing yielded an average of 8.1 million paired-end 150-bp reads per genome, providing 200× ± 7 mean depth coverage when aligned to the reference genome (Kmar_1.0, GCF_001417885.1). Reads were trimmed with Fastp v0.20.0 (7) with the option *-l* 50 and *de novo* assembled with SPAdes v3.12.0 (8) with default parameters, with no additional polishing applied. ITS and D1/D2 rDNA regions were extracted *in silico* from assemblies using universal oligos (9, 10) and compared with type strain CBS 712ᵀ (NCBI accessions KY103793.1 and KY108075.1), confirming the species taxonomy (99–100% identity). Assembly quality was assessed with QUAST v5.2.0 (11); six genomes showed similar quality, with 80–140 contigs and an N50 of ~440 kb. Genome length averaged 10.7 Mb with 40.08% GC content, consistent with the reference (10.9 Mb, 40% GC). One beer strain (UFSY-1180) had a more fragmented assembly (>200 contigs). BUSCO v5.6.1 (12) ran in genome mode against Saccharomycetes_odb10 (*n *= 2137 gene models) indicated >98% completeness. FastANI v1.34 with the option -*k* 20 bp in *k-mer* size (13) showed a considerably low average nucleotide identity (ANI) of the agave isolates with the reference genome strain DMKU3-1042 (~95.5% ANI, Table 1).

**TABLE 1:** Metadata and metrics of the seven *K. marxianus* genome assemblies from South Africa

Strain	Source	Year	Coverage (%)^*a*^	Depth (×)^*a*^	QUAST metrics				BUSCO assessment (%)					ANI (%)^*c*^	Assembly accession
					Contigs	Size (Mb)	GC (%)	*N_50_* (kb)	Complete^*b*^	Single	Duplicated	Fragmented	Missing		
UFSY-1180	Beer	1985	98	194	277	10.8	40.11	439	98.2	71.9	26.3	0.4	1.4	97.67	GCA_053524955.1
UFSY-1181	Beer	1989	98	208	80	10.7	40.06	441	98.4	72.3	26.1	0.6	1	97.66	GCA_053524915.1
UFSY-1182	Beer	1989	98	207	91	10.6	40.06	441	98.4	72.3	26.1	0.6	1	97.67	GCA_053524935.1
UFSY-1183	Beer	1987	98	199	93	10.7	40.07	477	98.6	72.5	26.1	0.5	0.9	97.66	GCA_053524895.1
UFSY-1186	Soil	1989	99	206	143	10.7	40.20	275	98.4	72	26.4	0.5	1.1	99.63	GCA_053524875.1
UFSY-2624	Agave	2005	97	191	116	10.7	40.02	592	98.3	72	26.3	0.4	1.3	95.53	GCA_053524835.1
UFSY-2791	Agave	2007	97	201	132	10.7	40.04	421	98.2	71.9	26.3	0.5	1.3	95.55	GCA_053524855.1

^*a*^ Coverage and depth parameters were estimated by aligning the reads to the reference genome Kmar_1.0, GCF_001417885.1.

^*b*^ BUSCO complete: Single plus duplicate genes.

^*c*^ ANI when comparing each genome with the DMKU3-1042 reference genomic sequence.

The drafts reported here provide opportunities to study genomic adaptations and domestication signatures in *K. marxianus*. The proportion of duplicated genes (Table 1) is particularly intriguing, as similar patterns in *S. cerevisiae* are linked to segmental duplications in industrial strains (14–16). These duplicated regions have been reported to occur on aneuploid chromosomes and are associated with genome decay during domestication in yeasts (17, 18). It is possible that these *K. marxianus* strains bear genomic hallmarks of domestication, but further genome analysis will be required to elucidate their evolutionary trajectories.

## Data Availability

Genomic data are deposited at the NCBI (BioProject PRJNA1294383) and the accession numbers for each assembly are provided in Table 1.

## References

[1] Rajkumar AS, Varela JA, Juergens H, Daran J-MG, Morrissey JP. 2019. Biological parts for Kluyveromyces marxianus synthetic biology. Front Bioeng Biotechnol 7:97. doi:10.3389/fbioe.2019.00097 31134195 PMC6515861

[2] Zhang M, Ruan Y, Hu Y, Huo Y, Wang M, Zhu Z, Fang Y. 2025. A novel high-temperature Kluyveromyces marxianus as a microbial cell factory host for sesquiterpene production. Biochem Eng J 220:109739. doi:10.1016/j.bej.2025.109739

[3] Ortiz-Merino RA, Varela JA, Coughlan AY, Hoshida H, da Silveira WB, Wilde C, Kuijpers NGA, Geertman J-M, Wolfe KH, Morrissey JP. 2018. Ploidy variation in Kluyveromyces marxianus separates dairy and non-dairy isolates. Front Genet 9:94. doi:10.3389/fgene.2018.00094 29619042 PMC5871668

[4] Heintz N, Gong S. 2020. Growth of Saccharomyces cerevisiae and preparation of DNA. Cold Spring Harb Protoc 2020:db. doi:10.1101/pdb.prot098145 33004551

[5] Li J, Zhai Z, Zhang H, Su Z, Liu Y, Chen H, Li Y, Shen M. 2024. Deep learning enables the use of ultra-high-density array in DNBSEQ. Sci Rep 14:27847. doi:10.1038/s41598-024-78748-x 39537672 PMC11561341

[6] Xu Y, Lin Z, Tang C, Tang Y, Cai Y, Zhong H, Wang X, Zhang W, Xu C, Wang J, Wang J, Yang H, Yang L, Gao Q. 2019. A new massively parallel nanoball sequencing platform for whole exome research. BMC Bioinformatics 20:153. doi:10.1186/s12859-019-2751-3 30909888 PMC6434795

[7] Chen S, Zhou Y, Chen Y, Gu J. 2018. fastp: an ultra-fast all-in-one FASTQ preprocessor. Bioinformatics 34:i884–i890. doi:10.1093/bioinformatics/bty560 30423086 PMC6129281

[8] Prjibelski A, Antipov D, Meleshko D, Lapidus A, Korobeynikov A. 2020. Using SPAdes de novo assembler. Curr Protoc Bioinformatics 70:e102. doi:10.1002/cpbi.102 32559359

[9] Kurtzman CP, Robnett CJ. 1998. Identification and phylogeny of ascomycetous yeasts from analysis of nuclear large subunit (26S) ribosomal DNA partial sequences. Antonie Van Leeuwenhoek 73:331–371. doi:10.1023/a:1001761008817 9850420

[10] Sylvester K, Wang Q-M, James B, Mendez R, Hulfachor AB, Hittinger CT. 2015. Temperature and host preferences drive the diversification of Saccharomyces and other yeasts: a survey and the discovery of eight new yeast species. FEMS Yeast Res 15:fov002. doi:10.1093/femsyr/fov002 25743785

[11] Gurevich A, Saveliev V, Vyahhi N, Tesler G. 2013. QUAST: quality assessment tool for genome assemblies. Bioinformatics 29:1072–1075. doi:10.1093/bioinformatics/btt086 23422339 PMC3624806

[12] Simão FA, Waterhouse RM, Ioannidis P, Kriventseva EV, Zdobnov EM. 2015. BUSCO: assessing genome assembly and annotation completeness with single-copy orthologs. Bioinformatics 31:3210–3212. doi:10.1093/bioinformatics/btv351 26059717

[13] Jain C, Rodriguez-R LM, Phillippy AM, Konstantinidis KT, Aluru S. 2018. High throughput ANI analysis of 90K prokaryotic genomes reveals clear species boundaries. Nat Commun 9:5114. doi:10.1038/s41467-018-07641-9 30504855 PMC6269478

[14] Liu ZL, Huang X. 2022. Copy number variants impact phenotype-genotype relationships for adaptation of industrial yeast Saccharomyces cerevisiae. Appl Microbiol Biotechnol 106:6611–6623. doi:10.1007/s00253-022-12137-0 36117206

[15] Hotta N, Kotaka A, Matsumura K, Sasano Y, Hata Y, Harada T, Sugiyama M, Harashima S, Ishida H. 2024. Effect of yeast chromosome II aneuploidy on malate production in sake brewing. J Biosci Bioeng 137:24–30. doi:10.1016/j.jbiosc.2023.10.007 37989703

[16] Gallone B, Steensels J, Prahl T, Soriaga L, Saels V, Herrera-Malaver B, Merlevede A, Roncoroni M, Voordeckers K, Miraglia L, Teiling C, Steffy B, Taylor M, Schwartz A, Richardson T, White C, Baele G, Maere S, Verstrepen KJ. 2016. Domestication and divergence of Saccharomyces cerevisiae beer yeasts. Cell 166:1397–1410. doi:10.1016/j.cell.2016.08.020 27610566 PMC5018251

[17] De Chiara M, Barré BP, Persson K, Irizar A, Vischioni C, Khaiwal S, Stenberg S, Amadi OC, Žun G, Doberšek K, Taccioli C, Schacherer J, Petrovič U, Warringer J, Liti G. 2022. Domestication reprogrammed the budding yeast life cycle. Nat Ecol Evol 6:448–460. doi:10.1038/s41559-022-01671-9 35210580

[18] Clarke MN, Marsoner T, Adell MAY, Ravichandran MC, Campbell CS. 2023. Adaptation to high rates of chromosomal instability and aneuploidy through multiple pathways in budding yeast. EMBO J 42:e111500. doi:10.15252/embj.2022111500 36530167 PMC10106982

